# Supplementary material for: Partially methylated domains are hypervariable in breast cancer and fuel widespread CpG island hypermethylation
Source: Nat Commun. 2019 Apr 15;10:1749. doi: 10.1038/s41467-019-09828-0 (PMC6465362; doi:10.1038/s41467-019-09828-0)
Supplement: Supplementary file 1 — Supplementary Information [file 41467_2019_9828_MOESM1_ESM.pdf]

Partially methylated domains are hypervariable in breast cancer and fuel widespread CpG island hypermethylation

Arie B. Brinkman *et al.*

SUPPLEMENTARY INFORMATION

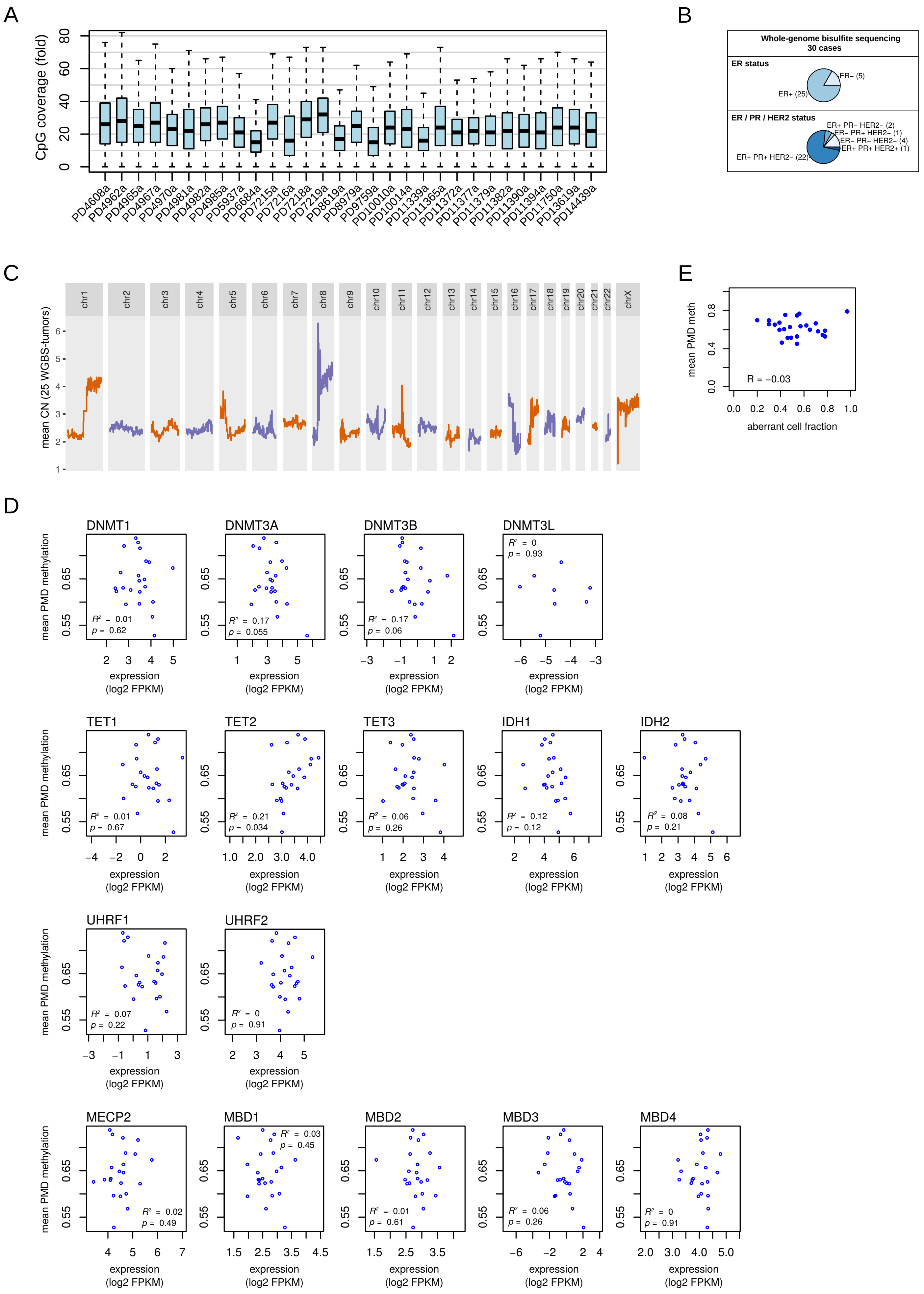

A
CpG coverage (fold)
0
20
40
60
80
PD4608a
PD4962a
PD4965a
PD4967a
PD4970a
PD4981a
PD4982a
PD4985a
PD5937a
PD6684a
PD7215a
PD7216a
PD7218a
PD7219a
PD8619a
PD8979a
PD9759a
PD10010a
PD10014a
PD11339a
PD11365a
PD11372a
PD11377a
PD11379a
PD11382a
PD11390a
PD11394a
PD11750a
PD13619a
PD14439a
B
Whole-genome bisulfite sequencing
30 cases
ER status
ER− (5)
ER+ (25)
ER / PR / HER2 status
ER+ PR− HER2− (2)
ER− PR+ HER2− (1)
ER− PR− HER2− (4)
ER+ PR+ HER2+ (1)
ER+ PR+ HER2− (22)
C
mean CN (25 WGBS-tumors)
chr1
chr2
chr3
chr4
chr5
chr6
chr7
chr8
chr9
chr10
chr11
chr12
chr13
chr14
chr15
chr16
chr17
chr18
chr19
chr20
chr21
chr22
chrX
E
mean PMD meth
R = −0.03
aberrant cell fraction
D
mean PMD methylation
expression
(log2 FPKM)
DNMT1
R^2 = 0.01
p = 0.62
DNMT3A
R^2 = 0.17
p = 0.055
DNMT3B
R^2 = 0.17
p = 0.06
DNMT3L
R^2 = 0
p = 0.93
TET1
R^2 = 0.01
p = 0.67
TET2
R^2 = 0.21
p = 0.034
TET3
R^2 = 0.06
p = 0.26
IDH1
R^2 = 0.12
p = 0.12
IDH2
R^2 = 0.08
p = 0.21
UHRF1
R^2 = 0.07
p = 0.22
UHRF2
R^2 = 0
p = 0.91
MECP2
R^2 = 0.02
p = 0.49
MBD1
R^2 = 0.03
p = 0.45
MBD2
R^2 = 0.01
p = 0.61
MBD3
R^2 = 0.06
p = 0.26
MBD4
R^2 = 0
p = 0.91

Supplementary Figure 1 | Sample characteristics: WGBS coverage, major pathological subtypes, copy-number profiles, expression of DNA methylation-related genes.

(A), CpG coverage in WGBS DNA methylation profiles of 30 breast tumor samples used in this study (see also Supplementary Table 1). Boxplots represent the median and 25th and 75th percentiles, whiskers 1.5 times the interquartile range, outliers are not shown. **(B)**, Clinicopathological features of the 30 tumor samples (see also Supplementary Table 2). **(C)**, Mean copy-number profiles of 25/30 tumor samples used in this study. Copy-number data was taken from our previous work[1]. **(D)**, Association between mean PMD methylation and expression of genes involved in writing, erasing, or reading the 5-methylcytosine modification. Each dot represents one tumor sample. Linear regression was used to determine the variation explained (R^2) and the p-value of the association. Expression data was taken from our previous work[2]. **(E)**, Mean PMD methylation (y-axis) is not associated with the fraction of aberrant cells (ASCAT[3], x-axis).

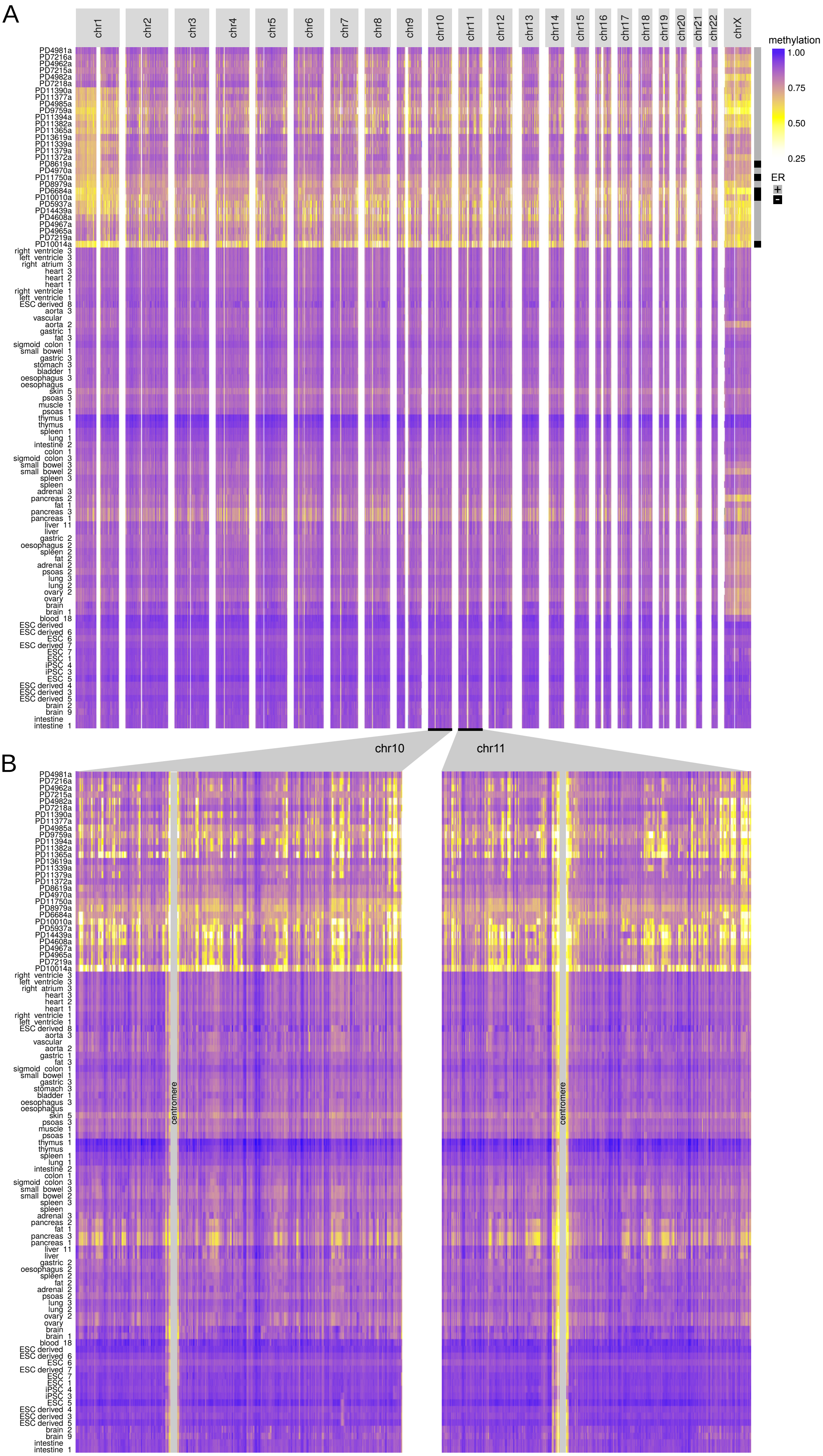

A
chr1
chr2
chr3
chr4
chr5
chr6
chr7
chr8
chr9
chr10
chr11
chr12
chr13
chr14
chr15
chr16
chr17
chr18
chr19
chr20
chr21
chr22
chrX
methylation
1.00
0.75
0.50
0.25
ER
+
-
PD4981a
PD7216a
PD4962a
PD7215a
PD4982a
PD7218a
PD11390a
PD11377a
PD4985a
PD9759a
PD11394a
PD11382a
PD11365a
PD13619a
PD11339a
PD11379a
PD11372a
PD8619a
PD4970a
PD11750a
PD8979a
PD6684a
PD10010a
PD5937a
PD14439a
PD4608a
PD4967a
PD4965a
PD7219a
PD10014a
right ventricle 3
left ventricle 3
right atrium 3
heart 3
heart 2
heart 1
right ventricle 1
left ventricle 1
ESC derived 8
aorta 3
vascular
aorta 2
gastric 1
fat 3
sigmoid colon 1
small bowel 1
gastric 3
stomach 3
bladder 1
oesophagus 3
oesophagus
skin 5
psoas 3
muscle 1
psoas 1
thymus 1
thymus
spleen 1
lung 1
intestine 2
colon 1
sigmoid colon 3
small bowel 3
small bowel 2
spleen 3
spleen
adrenal 3
pancreas 2
fat 1
pancreas 3
pancreas 1
liver 11
liver
gastric 2
oesophagus 2
spleen 2
fat 2
adrenal 2
psoas 2
lung 3
lung 2
ovary 2
ovary
brain
brain 1
blood 18
ESC derived
ESC derived 6
ESC 6
ESC derived 7
ESC 7
ESC 1
iPSC 4
iPSC 3
ESC 5
ESC derived 4
ESC derived 3
ESC derived 5
brain 2
brain 9
intestine
intestine 1
B
chr10
chr11
centromere
centromere

Supplementary Figure 2 | Visualization of inter-tumor variation at genome-wide scale, including non-tumor tissues.

Visualization of inter-tumor variation at genome-wide scale, as in main Figure 1, but including WGBS data from 72 additional, non-tumor tissues (Roadmap Epigenomics Project and ref.[4]). **(A)**, Genome-wide and **(B)**, chromosome-wide maps. Mean methylation is displayed in consecutive tiles of 10 kb (see Methods). For breast tumors of this study, the ER-status is indicated at the right (A).

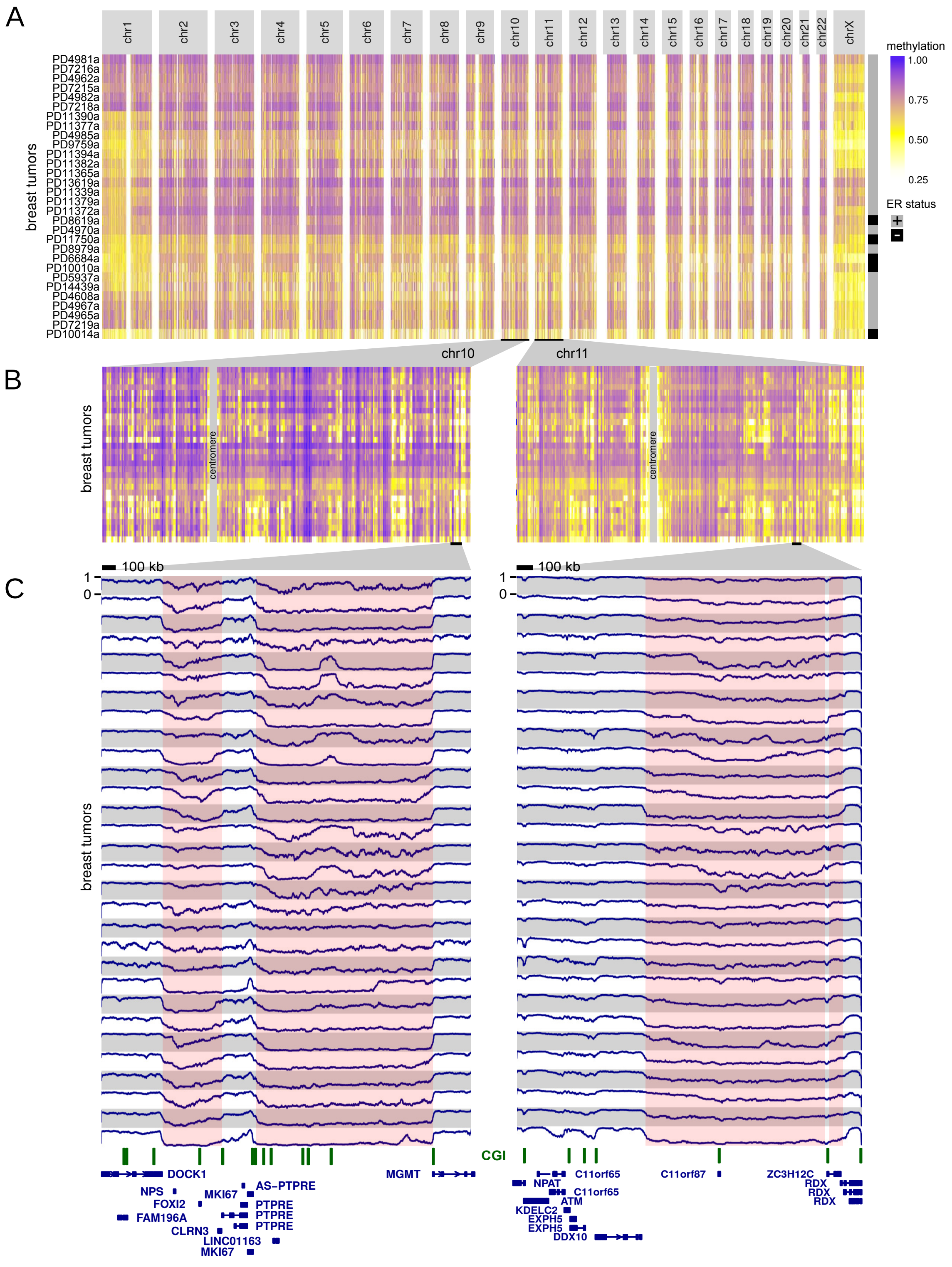

Supplementary Figure 3 | Visualization of inter-tumor variation at genome-wide scale, solo-WCGW CpGs only.

(A), Genome-wide and **(B)**, chromosome-wide maps of WGBS DNA methylation profiles from 30 breast tumor samples. Exactly as in Fig. 1AB, but using only solo-WCGW CpGs[5]. Mean methylation is displayed in consecutive tiles of 10 kb (see Methods). Ordering of tumor samples is the same as in Fig. 1. **(C)**, WGBS DNA methylation visualization at megabase-scale, exactly as in Fig. 1C, but using only solo-WCGW CpGs. Pink coloring indicates common methylation loss (PMDs) as in Fig. 1, although tumor-specific PMD borders vary. A scale bar (100 kb) is shown at the top of each panel. CpG islands are indicated in green.

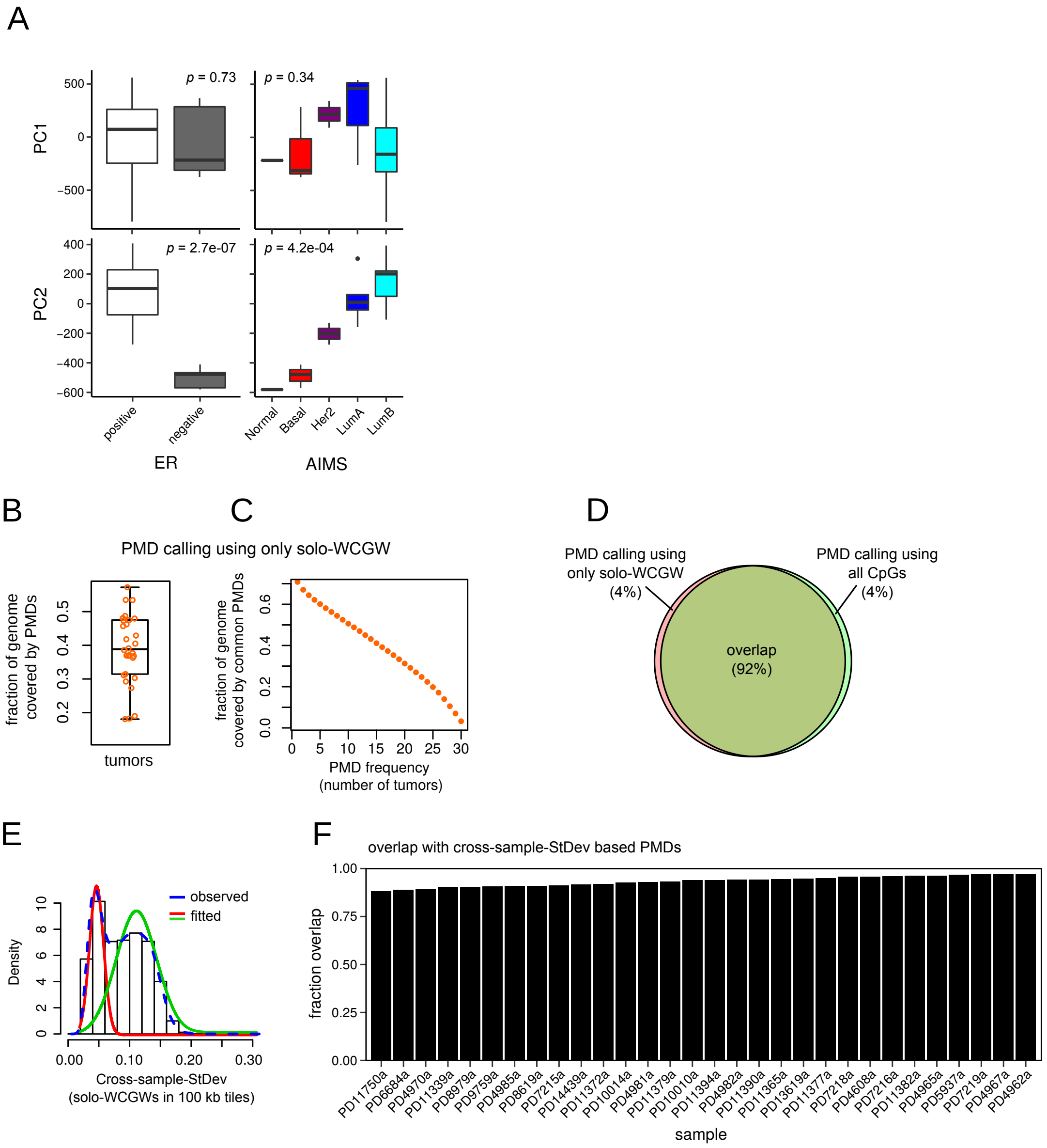

Supplementary Figure 4 | Association between DNA methylation variation and clinical features, PMD calling using solo-WCGW CpGs.

(A), Association between principal component 1 and 2 scores (PC1, PC2, see Fig. 1D) and major pathological subtypes. Significance testing was done as described in ref.[6]. Left panels, ER-status; right panels, AIMS intrinsic subtypes[7]. **(B,C)**, The same analysis as in Fig. 2AB, but using PMDs detected with only solo-WCGW CpGs[5]. Boxplots represent the median and 25th and 75th percentiles, whiskers 1.5 times the interquartile range. **(B)**, Fraction of the genome covered by PMDs. Each dot represents one tumor sample, the boxplot summarizes this distribution. **(C)**, Fraction of the genome covered by PMDs that are common between breast tumors. PMD frequency: the number of tumors in which a genomic region is a PMD. **(D)**, Venn-diagram showing the overlap between the union of all breast cancer PMDs ('all-CpGs') and the union of all breast cancer solo-WCGW PMDs. **(E)**, Bimodal distribution of cross-sample standard deviation of mean methylation in 100 kb genomic windows. Only solo-WCGW CpGs were used to calculate window means. As described in[5], a mixed gaussian was fitted to determine a cutoff for genome segmentation. **(F)**, Overlaps between the cross-sample s.d. based PMDs (E) and PMDs called on individual samples in this study, using all CpGs.

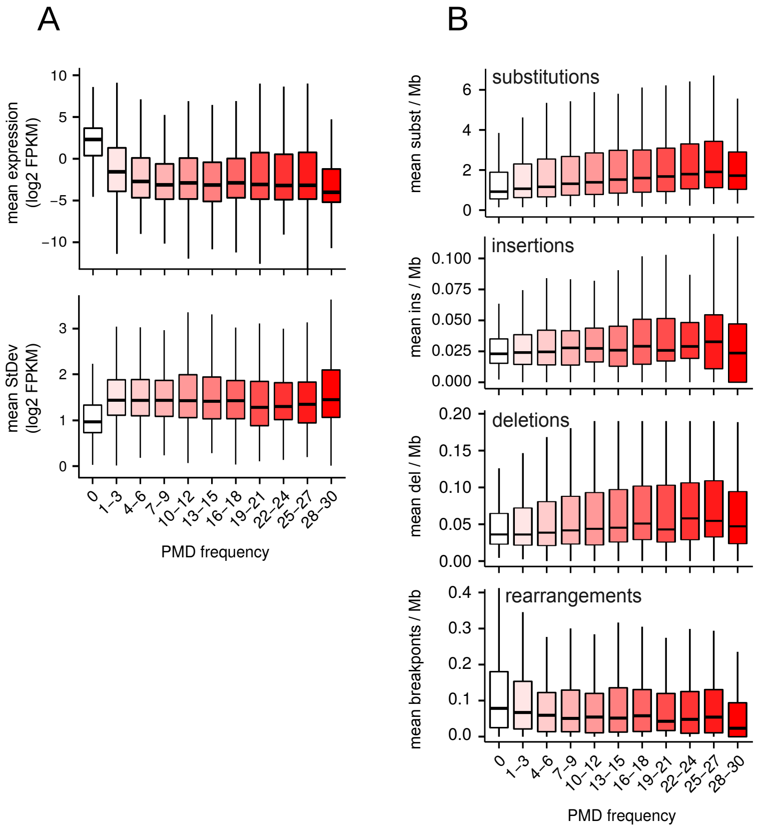

Supplementary Figure 5 | Gene expression and somatic mutations inside breast cancer PMDs, full cohort.

(A), Gene expression as a function of PMD frequency, as in main Figure 2F, but here extended to all 266 cases of the breast tumor (RNA-seq) transcriptomes cohort[2]. Top, gene expression; bottom, standard deviation. **(B)**, Somatic mutations plotted as a function of PMD frequency, as in main Figure 2G, but here extended to all 560 cases of the breast tumor full genomes cohort[1]. Boxplots represent the median and 25th and 75th percentiles, whiskers 1.5 times the interquartile range, outliers are not shown.

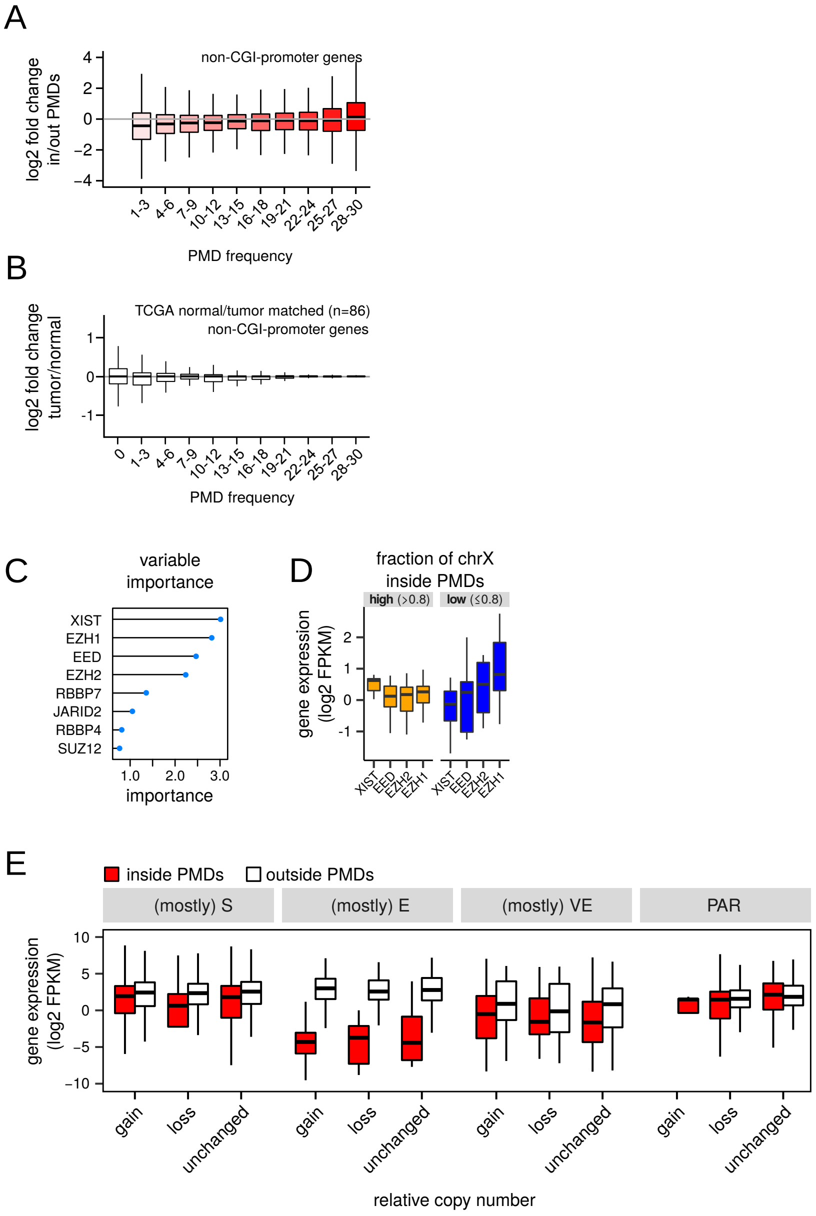

Supplementary Figure 6 | Expression change of non-CGI-promoter genes and X-linked genes inside PMDs, association of X-linked methylation loss with expression of PRC2 subunits.

(A), Expression change of non-CGI-promoter genes inside vs. outside of PMDs, as a function of PMD frequency. **(B)**, Expression change of non-CGI-promoter genes of tumor vs. normal, as a function of PMD frequency. From the TCGA breast cancer dataset, matched tumor/normal pairs were selected. PMD frequency for each gene was taken from our own dataset. **(C,D)**, Multivariate linear regression was performed with expression levels of genes involved in XCI as explanatory variables and PMD abundance on chrX as response variable. The variable importance of each XCI gene is plotted in (C), and their expression levels in two PMD abundance bins is plotted in (D). **(E)**, Expression of X-linked genes when inside or outside PMDs. Genes were grouped according their consensus X-inactivation status (E, escape; S, subject to XCI; VE, variably escaping; PAR, pseudoautosomal region)[8] and further stratified over their copy-number status (gain, loss, unchanged) as determined previously[1]. All boxplots in this figure represent the median and 25th and 75th percentiles, whiskers 1.5 times the interquartile range, outliers are not shown.

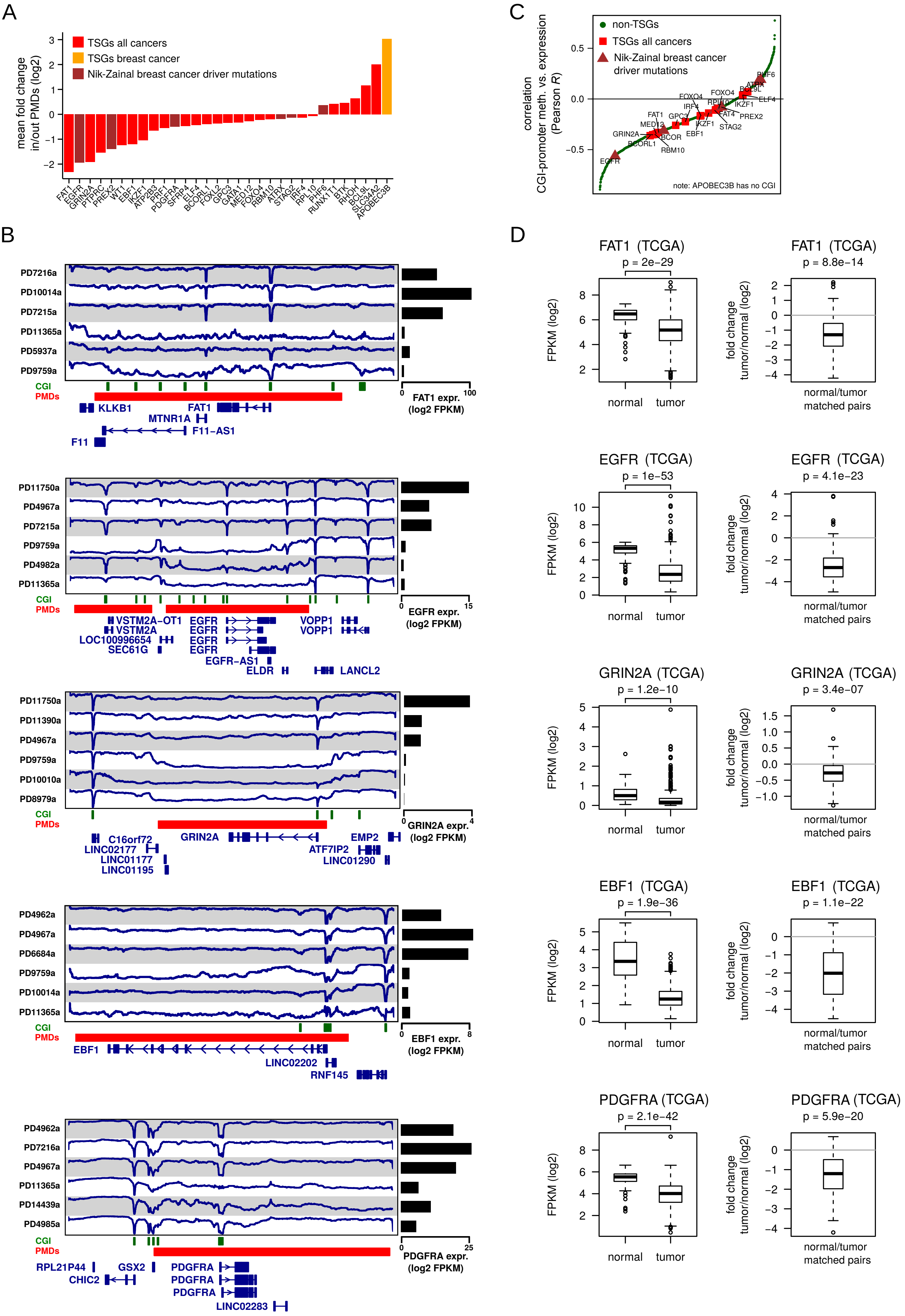

A
TSGs all cancers
TSGs breast cancer
Nik-Zainal breast cancer driver mutations
mean fold change in/out PMDs (log2)
FAT1 EGFR GRIN2A PTPRC PREX2 WT1 EBF1 IKZF1 ATP2B3 PRF1 PDGFRA SFRP4 ELF4 BCORL1 FOXL2 GPC3 GATA1 MED12 FOXO4 RBM10 ATRX STAG2 IRF4 RPL10 PHF6 RUNX1T1 BTK RHOH BCL9L SLC34A2 APOBEC3B
B
CGI
PMDs
KLKB1
FAT1
MTNR1A
F11-AS1
F11
FAT1 expr. (log2 FPKM)
EGFR expr. (log2 FPKM)
VSTM2A-OT1
VSTM2A
LOC100996654
SEC61G
EGFR
EGFR-AS1
ELDR
VOPP1
LANCL2
GRIN2A expr. (log2 FPKM)
C16orf72
LINC02177
LINC01177
LINC01195
GRIN2A
ATF7IP2
LINC01290
EMP2
EBF1 expr. (log2 FPKM)
EBF1
LINC02202
RNF145
PDGFRA expr. (log2 FPKM)
RPL21P44
GSX2
CHIC2
PDGFRA
LINC02283
C
non-TSGs
TSGs all cancers
Nik-Zainal breast cancer driver mutations
correlation CGI-promoter meth. vs. expression (Pearson R)
note: APOBEC3B has no CGI
D
FAT1 (TCGA)
p = 2e−29
p = 8.8e−14
EGFR (TCGA)
p = 1e−53
p = 4.1e−23
GRIN2A (TCGA)
p = 1.2e−10
p = 3.4e−07
EBF1 (TCGA)
p = 1.9e−36
p = 1.1e−22
PDGFRA (TCGA)
p = 2.1e−42
p = 5.9e−20
FPKM (log2)
fold change tumor/normal (log2)
normal
tumor
normal/tumor matched pairs

Supplementary Figure 7 | Expression change of tumor suppressor genes and breast cancer driver mutated genes inside PMDs.

(A), Expression change of TSGs/breast cancer driver mutated genes when inside PMDs. 31 of such genes are located inside PMDs in a subset of tumor samples. 'TSGs all cancers', genes annotated as TSGs regardless of cancer type; 'TSGs breast cancer', genes annotated as TSG in breast cancer; 'Nik-Zainal breast cancer driver mutations', genes with driver mutations in breast cancer[1]. **(B)**, Examples of genes from panel (A) being repressed when inside PMDs. Blue line, DNA methylation (WGBS); green bars, CGIs; red bars, PMDs. Gene expression (RNA-seq) of the corresponding gene is represented at the right of each panel. **(C)**, Pearson correlation between CGI-promoter methylation and expression. Gene classes are indicated as in panel (A). **(D)**, Expression changes (RNA-seq) of genes in panel (B), breast tumor vs. normal. Data is from an independent cohort (TCGA). Left panels, non-matched normal (n=88) and tumor samples (n=769); right panels, matched normal/tumor samples (n=86). p-values were calculated using a *t*-test. Boxplots represent the median and 25th and 75th percentiles, whiskers 1.5 times the interquartile range.

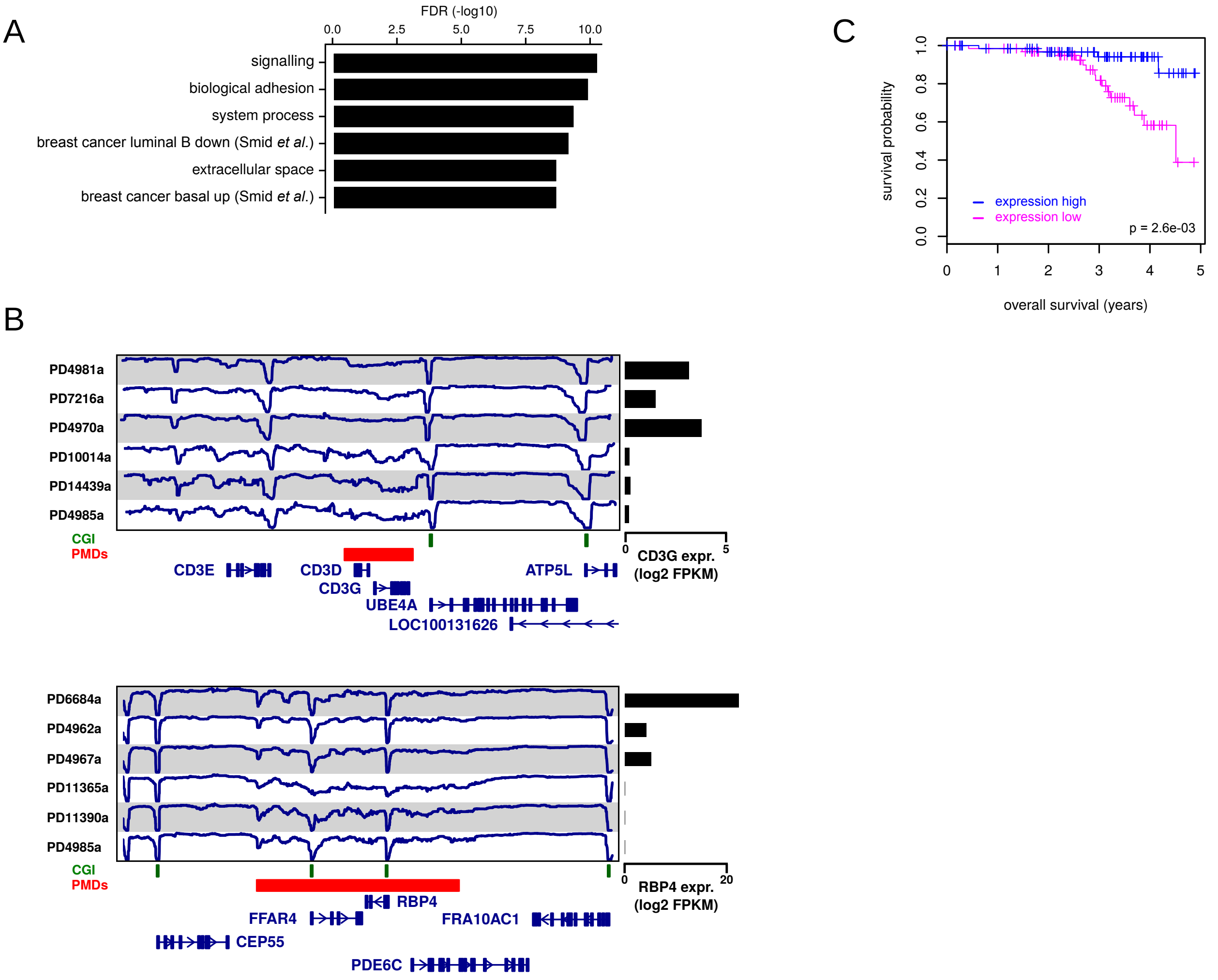

Supplementary Figure 8 | Gene set enrichment analysis (GSEA) of genes downregulated when inside PMDs.

(A), Gene set enrichment analysis (GSEA) of genes downregulated when inside PMDs (>2.5 log2-fold, 400 genes, Supplementary Table 4). **(B)**, Examples of downregulated genes inside PMDs. CD3D encodes the gamma polypeptide of the T-cell receptor-CD3 complex (gene sets ‘signalling’, ‘adhesion’, and ‘breast cancer luminal B down’); RBP4 encodes retinol binding protein 4 (gene set ‘signalling’, and ‘breast cancer luminal B down’). Blue line, DNA methylation (WGBS); green bars, CGIs; red bars, PMDs. Gene expression (RNA-seq) of the corresponding gene is represented at the right of each panel. **(C)**, Overall survival of patient groups stratified according expression of the 400 PMD-downregulated genes (see Methods).

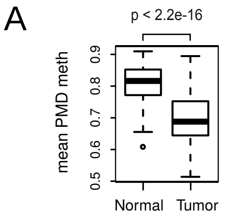

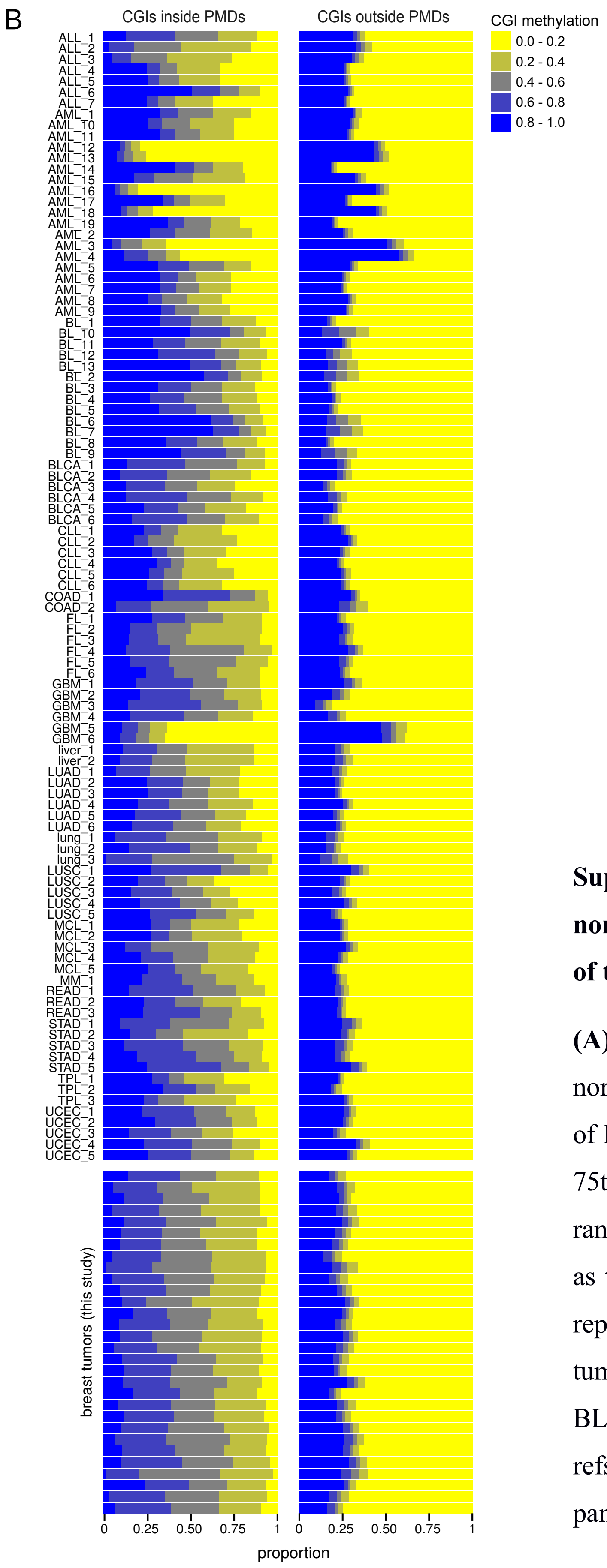

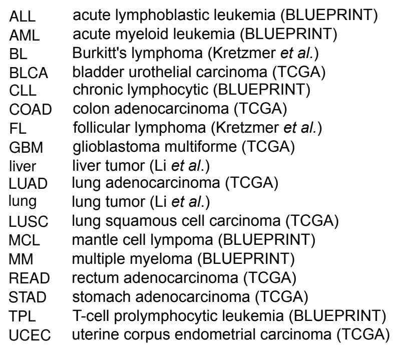

Supplementary Figure 9 | Mean PMD methylation of normal tissues and tumors, CpG island methylation of tumors from various tissues.

(A), Boxplot summarizing mean PMD methylation of normal tissues and tumors of various tissues (summary of Fig. 4B). Boxplots represent the median and 25th and 75th percentiles, whiskers 1.5 times the interquartile range. **(B)**, Distribution of CGI methylation, represented as the fraction of all CGIs (x-axis). Each horizontal bar represents one tumor sample (WGBS). Top panel, tumor samples other than breast cancer (TCGA, BLUEPRINT, the Roadmap Epigenomics Project, refs.[4,9,10], abbreviations are given on the right); bottom panel, repeated from main Figure 3B for comparison.

SUPPLEMENTARY REFERENCES

1. Nik-Zainal, S. *et al.* Landscape of somatic mutations in 560 breast cancer whole-genome sequences. *Nature* **534,** 1–20 (2016).

2. Smid, M. *et al.* Breast cancer genome and transcriptome integration implicates specific mutational signatures with immune cell infiltration. *Nature Communications* **7,** 12910 (2016).

3. Van Loo, P. *et al.* Allele-specific copy number analysis of tumors. *Proceedings of the National Academy of Sciences* **107,** 16910–16915 (2010).

4. Schultz, M. D. *et al.* Human body epigenome maps reveal noncanonical DNA methylation variation. *Nature* **523,** 212–6 (2015).

5. Zhou, W. *et al.* DNA methylation loss in late-replicating domains is linked to mitotic cell division. *Nature Genetics* **50,** 591–602 (2018).

6. Le, S., Josse, J. & Husson, F. FactoMineR: An R Package for Multivariate Analysis. *J. of Statistical Software* **25,** 1–18 (2008).

7. Sorlie, T. *et al.* Repeated observation of breast tumor subtypes in independent gene expression data sets. *Proceedings of the National Academy of Sciences of the United States of America* **100,** 8418–8423 (2003).

8. Balaton, B. P., Cotton, A. M. & Brown, C. J. Derivation of consensus inactivation status for X-linked genes from genome-wide studies. *Biology of Sex Differences* **6,** 35 (2015).

9. Kretzmer, H. *et al.* DNA methylome analysis in Burkitt and follicular lymphomas identifies differentially methylated regions linked to somatic mutation and transcriptional control. *Nature genetics* **47,** 1316–25 (2015).

10. Li, X., Liu, Y., Salz, T., Hansen, K. D. & Feinberg, A. Whole genome analysis of the methylome and hydroxymethylome in normal and malignant lung and liver. *Genome Research* **26,** 1730–1741 (2016).
