# Supplementary material for: Partially methylated domains are hypervariable in breast cancer and fuel widespread CpG island hypermethylation
Source: Nat Commun. 2019 Apr 15;10:1749. doi: 10.1038/s41467-019-09828-0 (PMC6465362; doi:10.1038/s41467-019-09828-0)
Supplement: Supplementary file 3 — Description of Additional Supplementary Files [file 41467_2019_9828_MOESM3_ESM.pdf]

Description of Additional Supplementary Information

File Name: Supplementary Data 1
Description: Quality metrics and global methylation values from whole-genome bisulfite.

File Name: Supplementary Data 2
Description: Clinical and molecular data of the 30 breast tumor samples analyzed by whole-genome bisulfite sequencing.

File Name: Supplementary Data 3
Description: PMD frequency of all annotated CpG islands.

File Name: Supplementary Data 4
Description: Genes that are downregulated at least 2.5 log2-fold when inside PMDs.

File Name: Supplementary Data 5
Description: External data used in this study.
