# Supplementary material for: Partially methylated domains are hypervariable in breast cancer and fuel widespread CpG island hypermethylation
Source: Nat Commun. 2019 Apr 15;10:1749. doi: 10.1038/s41467-019-09828-0 (PMC6465362; doi:10.1038/s41467-019-09828-0)
Supplement: Supplementary file 9 — Reporting Summary [file 41467_2019_9828_MOESM9_ESM.pdf]

nature research

Corresponding author(s): H.G. Stunnenberg, A.B. Brinkman

Last updated by author(s): Mar 13, 2019

Reporting Summary

Nature Research wishes to improve the reproducibility of the work that we publish. This form provides structure for consistency and transparency in reporting. For further information on Nature Research policies, see Authors & Referees and the Editorial Policy Checklist.

Statistics

For all statistical analyses, confirm that the following items are present in the figure legend, table legend, main text, or Methods section.

n/a	Confirmed	
☐	☒	The exact sample size (*n*) for each experimental group/condition, given as a discrete number and unit of measurement
☒	☐	A statement on whether measurements were taken from distinct samples or whether the same sample was measured repeatedly
☐	☒	The statistical test(s) used AND whether they are one- or two-sided<br>*Only common tests should be described solely by name; describe more complex techniques in the Methods section.*
☒	☐	A description of all covariates tested
☒	☐	A description of any assumptions or corrections, such as tests of normality and adjustment for multiple comparisons
☐	☒	A full description of the statistical parameters including central tendency (e.g. means) or other basic estimates (e.g. regression coefficient) AND variation (e.g. standard deviation) or associated estimates of uncertainty (e.g. confidence intervals)
☐	☒	For null hypothesis testing, the test statistic (e.g. *F*, *t*, *r*) with confidence intervals, effect sizes, degrees of freedom and *P* value noted<br>*Give P values as exact values whenever suitable.*
☒	☐	For Bayesian analysis, information on the choice of priors and Markov chain Monte Carlo settings
☒	☐	For hierarchical and complex designs, identification of the appropriate level for tests and full reporting of outcomes
☐	☒	Estimates of effect sizes (e.g. Cohen's *d*, Pearson's *r*), indicating how they were calculated

Our web collection on statistics for biologists contains articles on many of the points above.

Software and code

Policy information about availability of computer code

Data collection

Whole -genome bisulfite read mapping and methylation calling was done using the 'rmapbs-pe' tool from the MethPipe v3.0.0 package, and 'duplicate-remover', 'methcounts', tools from the MethPipe v2.03 software package.
For all analyses throughout this study the GRCh37/hg19 human genome build was used

Data analysis

All code for analyses of this study is available on https://github.com/abbrinkman/brcancer_wgbs.git.

Software versions used:
R (v3.4.3) was used for all analyses, while making use of functionality provided in the following packages:
betareg (v3.1-0)
biomaRt (v2.34.2)
BSgenome.Hsapiens.UCSC.hg19 (v1.4.0)
caret (v6.0-78)
circlize (v0.4.3)
cluster (v2.0.6)
ComplexHeatmap (v1.17.1)
data.table (v1.10.4-3)
FactoMineR (v1.39)
FDb.UCSC.snp137common.hg19 (v1.0.0)
gdata (v2.18.0)
GenomicRanges (v1.30.2)
ggdendro (v0.1-20)
ggplot2 (v2.2.1)
ggrepel (v0.7.0)
ggthemes (v3.4.0)
gplots (v3.0.1)

gridExtra (v2.3)
Gviz (v1.22.2)
lme4 (v1.1-15)
matrixStats (v0.53.1)
MethylSeekR (v1.18.0)
missMDA (v1.11)
openxlsx (v4.0.17)
plyr (v1.8.4)
qvalue (v2.10.0)
RColorBrewer (v1.1-2)
reshape2 (v1.4.3)
rtracklayer (v1.38.3)
snow (v0.4-2)
stringr (v1.2.0)
TxDb.Hsapiens.UCSC.hg19.knownGene (v3.2.2)
cluster (v2.0.6)
grid (v3.4.3)
MASS (v7.3-48)
parallel (v3.4.3)
dplyr (v0.7.8)
forcats (v0.3.0)
purrr (v0.3.0)
readr (v1.3.1)
stringr (v1.4.0)
tibble (v2.0.1)
tidyr (v0.8.2)

For manuscripts utilizing custom algorithms or software that are central to the research but not yet described in published literature, software must be made available to editors/reviewers. We strongly encourage code deposition in a community repository (e.g. GitHub). See the Nature Research guidelines for submitting code & software for further information.

Data

Policy information about availability of data

All manuscripts must include a data availability statement. This statement should provide the following information, where applicable:

- Accession codes, unique identifiers, or web links for publicly available datasets
- A list of figures that have associated raw data
- A description of any restrictions on data availability

Data availability statement:
Tables containing CpG methylation values (bigwig), genomic coordinates and mean methylation values of PMDs and CGIs are available via DOI 10.5281/zenodo.1467025 or DOI 10.17026/dans-276-sda6. Raw data for whole-genome bisulfite sequencing of the 30 breast tumor samples of this study is available from the European Genome-phenome Archive (https://www.ebi.ac.uk/ega) under dataset accession EGAD00001001388 (Study EGAS00001001195, Data Access Committee EGAC00001000010). External data resources used in this study are listed in Supplementary Table 5.

Figures that have associated raw data:
Figures with associated raw data from this study
Figure 1ABCD
Figure 2ABCDEFGH
Figure 3ABCDEFGHKL
Figure 4AB
Supplementary Figure 1ABDE
Supplementary Figure 2AB
Supplementary Figure 3AB
Supplementary Figure 4BCDEF
Supplementary Figure 5AB
Supplementary Figure 6ACDE
Supplementary Figure 7ABC
Supplementary Figure 8ABC

Restrictions on data availabilty
All data is publicly available, except raw data (FASTQ files with sequencing reads), and tables describing read coverage for each CG in the genome. These restrictions apply because this is regarded as identifyable data. This part of the data is available through controlled access (Data Access Comittee) in the European Genome-phenome Archive (https://www.ebi.ac.uk/ega, see above for the accession number)

Field-specific reporting

Please select the one below that is the best fit for your research. If you are not sure, read the appropriate sections before making your selection.

☒ Life sciences ☐ Behavioural & social sciences ☐ Ecological, evolutionary & environmental sciences

For a reference copy of the document with all sections, see nature.com/documents/nr-reporting-summary-flat.pdf

Life sciences study design

All studies must disclose on these points even when the disclosure is negative.

Sample size	30
Data exclusions	None
Replication	As these are tumor samples from human individuals, no replicates are available
Randomization	No randomization was involved
Blinding	Blinding was not relevant, as no grouping was involved

Reporting for specific materials, systems and methods

We require information from authors about some types of materials, experimental systems and methods used in many studies. Here, indicate whether each material, system or method listed is relevant to your study. If you are not sure if a list item applies to your research, read the appropriate section before selecting a response.

Materials & experimental systems

n/a	Involved in the study	
☒	☐	Antibodies
☒	☐	Eukaryotic cell lines
☒	☐	Palaeontology
☒	☐	Animals and other organisms
☒	☐	Human research participants
☐	☒	Clinical data

Methods

n/a	Involved in the study	
☒	☐	ChIP-seq
☒	☐	Flow cytometry
☒	☐	MRI-based neuroimaging

Clinical data

Policy information about clinical studies

All manuscripts should comply with the ICMJE guidelines for publication of clinical research and a completed CONSORT checklist must be included with all submissions.

Clinical trial registration	Not applicable: This study is an analysis of human tumor samples provided by multiple hospitals/tissue banks/research institutes, not as part of a clinical trial
Study protocol	Not applicable
Data collection	Not applicable
Outcomes	Not applicable
